# COVID-19 Impact on Adolescent 24 h Movement Behaviors

**DOI:** 10.3390/ijerph18179256

**Published:** 2021-09-02

**Authors:** Marie-Maude Dubuc, Félix Berrigan, Marylène Goudreault, Sylvie Beaudoin, Sylvain Turcotte

**Affiliations:** 1 Faculté des Sciences de l’Activité Physique, Université de Sherbrooke, Sherbrooke, QC J1K 2R1, Canada; felix.berrigan@usherbrooke.ca (F.B.); sylvie.beaudoin@usherbrooke.ca (S.B.); sylvain.turcotte@usherbrooke.ca (S.T.); 2 Kino-Québec Research Chair on the Adoption of a Physically Active Lifestyle in School Contexts, Sherbrooke, QC J1K 2R1, Canada; 3 Direction Régionale de Santé Publique du CIUSSS du Centre-Sud-de-l’île-de-Montréal, Montréal, QC H2L 4M1, Canada; marylene.goudreault.ccsmtl@ssss.gouv.qc.ca

**Keywords:** physical activity, screen time, sleep, sedentary behavior, secondary school, technology

## Abstract

This study aimed to examine the impact of the COVID-19 pandemic on the 24 h movement behaviors of adolescents. This was conducted to capture their evolution from February to December 2020, as well as to explore the use of technology for physical activity purposes by adolescents as a strategy to increase their physical activity during the pandemic. Physical activity, recreational screen time, sleep duration, and sleep quality were self-reported by 2661 adolescents using an online questionnaire. Participants also indicated, in comparison with the previous winter (regular in-class learning), how their different movement behaviors changed during the following 2020 periods: (1) spring (school closures), (2) summer (school break), and (3) autumn (hybrid learning). Finally, information about the use of technology during physical activity was collected. Results show that the 24 h movement behaviors of the participants varied across the different periods, and these variations were consistent with the restrictive measures imposed by the government. It was also observed that the negative effects of the COVID-19 pandemic on sleep duration and quality peaked in autumn. Finally, participants’ physical activity levels were associated with the use of physical activity-related tools and applications. In conclusion, the restrictive measures due to the COVID-19 pandemic worsened the situation of the 24 h movement behaviors in adolescents, which has become critical.

## 1. Introduction

The Canadian 24 h movement guidelines [1] promote the importance of adopting an active lifestyle by addressing physical activity (PA) practice, sedentary behavior, and sleep habits. During adolescence, it is recommended to perform at least 60 min per day of moderate to vigorous PA, to limit recreational screen time to a maximum of 2 h per day, and to sleep steadily between 9 and 11 h per night (12–13 years old) or between 8 and 10 h per night (14–17 years old) [1]. It has been previously reported in Canada that children and adolescents struggle to follow these guidelines, with less than 20% of them successfully respecting these three recommendations [2,3].

The COVID-19 pandemic has led the three main levels of Canadian governments, namely federal, provincial, and municipal governments, to impose restrictive measures such as border closures, social distancing, quarantining, school closures, and more. As of the end of February 2021, one of the most impacted regions in Canada remains the Montreal area, with slightly more than 12% of the confirmed cases of infected persons in the country. In this area, adolescents experienced distance learning from 13 March 2020 until the end of June 2020 (summer break). During that period, a lockdown was imposed, forcing organized sports to stop or to continue virtually. Thereafter, restrictive measures decreased with the upcoming summer, allowing adolescents to practice organized sports with respect to various Public Health’s measures to reduce the spread of the virus. In September 2020, adolescents from grades 7 to 11 (12 to 17 years old) were back in schools. However, a few weeks later, hybrid education, a mix of in-person and online activities, was provided to older students (grades 9 to 11; 14 to 17 years old). Most of the extracurricular activities and organized sports were forced to stop or to continue virtually once again.

These restrictive measures may have exacerbated adolescents’ difficulties to follow the 24 h movement guidelines [3,4]. For example, preliminary evidence from a Canadian study conducted in parents of 1472 children and youth (5–17 years old) suggested that these measures had an adverse impact on youth PA levels, sedentary behaviors, and sleep [4]. Indeed, in the adolescent’s subgroup (12–17 years old; *n* = 774), it was reported that only 0.6% of the 12–17 years old met the three recommendations of the 24 h movement guidelines during the lockdown of April 2020 [4]. However, little is known about the evolution of adolescents’ movement behaviors during the months following the spring 2020 lockdown. Furthermore, there is limited information about the strategies developed by adolescents during the pandemic to practice PA. Nevertheless, it was reported that the use of technology for PA purposes was positively associated with moderate to vigorous PA in adolescents and adults during the pandemic [5,6].

Adolescents already represent a population struggling to adopt healthy movement behaviors, and the restrictions due to the COVID-19 pandemic appear to decrease their propensity to succeed in adopting healthy movement behaviors. Therefore, it seemed relevant to examine the impact of these restrictions on the 24 h movement of adolescents in one of the most affected communities in Canada. Thus, the present study aimed to examine the impact of the COVID-19 pandemic on PA practice, sedentary behavior associated with recreational screen time, and sleep habits of adolescents, to capture their evolution from February to December 2020 as well as to explore the use of technology for PA purposes as a strategy to increase their PA practice during the pandemic.

## 2. Materials and Methods

### 2.1. Overview

The results of this study are based on data collected within a collaborative research project involving University of Sherbrooke researchers, the Montreal Public Health, the school boards of the Montreal area, the City of Montreal, as well as public organizations engaged in the promotion of a physically active lifestyle in adolescents. Seventeen public secondary schools belonging to the school boards of the Montreal area accepted to participate in the study. Inclusion criteria for schools were: (1) to offer the regular secondary education program of the province of Quebec and (2) to serve grades 7 to 11 students, which represents the complete secondary education curriculum in the province. Seven of the participating secondary schools were English-language schools, while 10 of them were French-language schools. The data collection was performed during December 2020. 

### 2.2. Participants

The study sample consisted of 2661 secondary school students, which included 1417 girls (53.3%), 1154 boys (43.4%), 33 gender-diverse (1.2%), and 57 participants who did not indicate their gender (2.1%), representing a 69% response rate. Inclusion criterion for participants was to be a regular student of one of the seventeen participating schools.

### 2.3. Questionnaire

**Figure 1:**
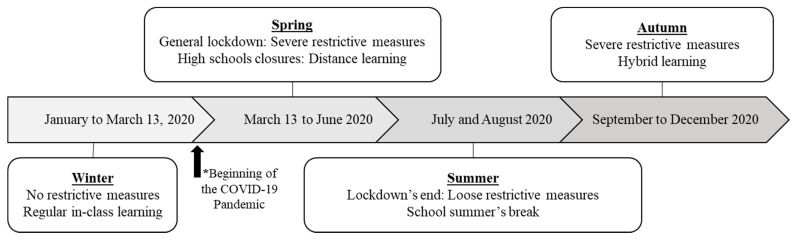
Timeline of the year 2020 in regard to the COVID-19 restrictive measures and the schooling situation. * In the Montreal area, restrictive measures due to the COVID-19 pandemic started on 13 March 2020.

All participants completed a 30 min online questionnaire (filled out either in English or French) during one of their classroom periods (virtual or in-class). Within the questionnaire, they were asked to qualitatively compare their movement behaviors (PA practice, screen recreational screen time, and sleep duration) during different periods of 2020 to “prior to the spring’s lockdown period (before 13 March 2020)”, referring to the last regular in-class learning period. Figure 1 presents the characteristics of these four different periods of 2020, which will thereafter be named winter, spring, summer, and autumn. Participants indicated if their PA practice, recreational screen time (excluding school-related screen time), and sleep duration varied during the spring, summer, and autumn in comparison to the previous winter on a five-level scale (from “decreased significantly” to “increased significantly”). They also indicated, using a question extracted from the Pittsburgh Sleep Quality Index [7], their sleep quality on a 4-point scale (from “very bad” to “very good”) for each of the four periods of 2020 previously described. Thereafter, participants reported their actual PA practice (excluding physical education and health class) and recreational screen time in minutes per day for both weekdays and weekends as of December 2020. Using another question extracted from the Pittsburgh Sleep Quality Index [7], participants reported their actual sleep duration in hours per night for both weekdays and weekends. Finally, participants indicated if they had used technology when practicing PA over the last month, excluding in the physical education and health class context. When applicable, they specified what tool(s) or application(s) they used and the reason(s) why they used it (them). Thereafter, PA tools and applications were regrouped using the following categories: training exercises, pedometer, general health, weight loss, exercise inspiration, watches, tracking, entertainment during PA, and others.

### 2.4. Statistical Analysis

Descriptive statistics were calculated for all study variables. A two-proportion bilateral z-test was performed to assess proportional differences in PA practice, recreational screen time, sleep duration, and sleep quality over the different periods of the 2020 year. Throughout the analyses, gender differences were assessed using chi square tests. Chi square tests were also performed to explore the association between the use of PA-related technology and the actual PA practice of participants. Significance was defined at *p* < 0.05. Thereafter, 95% confidence intervals were also calculated to estimate the proportion of adolescents following the 24 h movement guidelines as of December 2020. Finally, a one-proportion bilateral z-test was performed to assess differences in the proportion of adolescents following the PA recommendations, the recreational screen time recommendations, and the sleep duration recommendations between the autumn 2020 and prior the COVID-19 pandemic (using data collected from a previous study by Roberts et al. [2]). Statistical analysis was performed using SPSS 27 for Windows (IBM Corp., New York, NY, USA).

## 3. Results

### 3.1. Evolution of the 24 h Movement Behaviors

**Figure 2:**
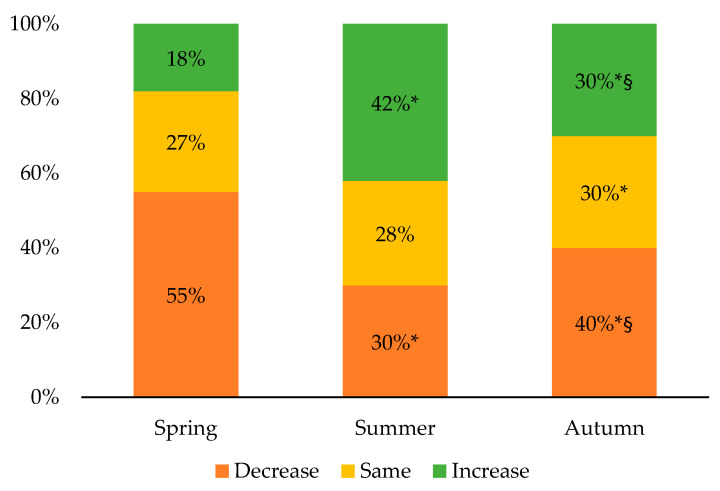
Evolution of physical activity practice compared to the winter 2020. * Significantly different from the spring; ^§^ Significantly different from the summer; *p* < 0.01.

Most of the participants (55%) reported a decrease in their PA practice during the spring of either a slight decrease (29%) or a significant decrease (26%), as shown in Figure 2. During that same period, 18% of the participants reported an increase in their PA practice of either a slight increase (11%) or a significant increase (7%). In the summer, 30% of the participants reported a decrease in their PA practice in comparison with the winter (21% reported a slight decrease and 9% a significant decrease), while 42% of them reported an increase (28% reported a slight increase and 14% a significant increase). In the autumn, 40% of the participants experienced a decrease in their PA practice compared to the winter, with 25% of them reporting a slight decrease and 15% a significant decrease. Over that same period, 30% of the participants reported an increase in their PA practice compared to the winter, with either a slight increase (22%) or a significant increase (8%). It should be noted that a significant gender effect was only observed during the spring, while a larger proportion of girls reported experiencing changes in their PA practice than boys or gender-diverse participants (77%, 71%, and 61%, respectively; *p* < 0.001).

**Figure 3:**
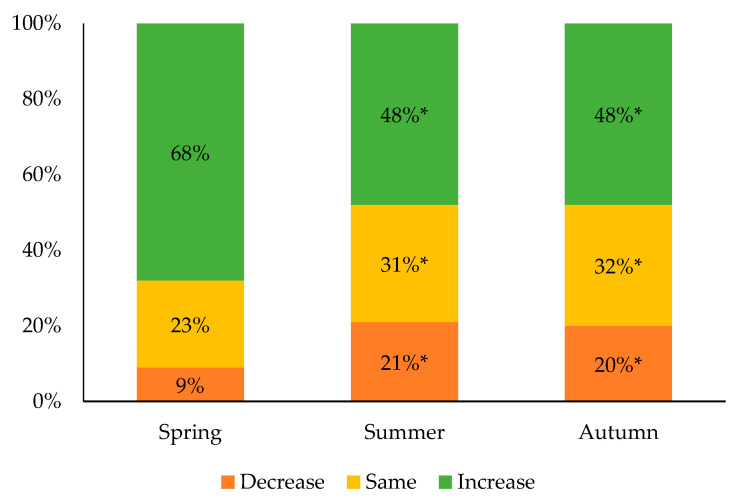
Evolution of recreational screen time compared to the winter 2020. * Significantly different from the spring; *p* < 0.001.

Figure 3 presents the evolution of the participants’ recreational screen time from the spring to the autumn in comparison to the winter. Most of the participants had increased their recreational screen time during the spring (68%), reporting either a slight increase (25%) or a significant increase (43%). Few participants (9%) reported a decrease in their recreational screen time during that same period, with 5% of them reporting a slight decrease and 4% a significant decrease. A significant gender effect was also observed during the spring, while a greater proportion of girls and gender-diverse participants reported experiencing an increased recreational screen time than boys (72%, 71%, and 63%, respectively; *p* < 0.001). In the summer, 48% of the participants reported an increase (24% reported a slight increase and 24% a significant increase), while 21% of them reported a decrease in their recreational screen time in comparison with the winter (16% reported a slight decrease and 5% a significant decrease). Almost half of the participants (48%) were still reporting an increased recreational screen time in the autumn compared to the winter, with 25% of them reporting a slight increase and 23% a significant increase. Over that same period, 20% of the participants reported a decrease in their recreational screen time compared to the winter, as either a slight decrease (14%) or a significant decrease (6%). During both the summer and the autumn, a larger proportion of girls and gender-diverse participants reported continuing to experiment with more recreational screen time than during the winter (both *p* = 0.001).

**Figure 4:**
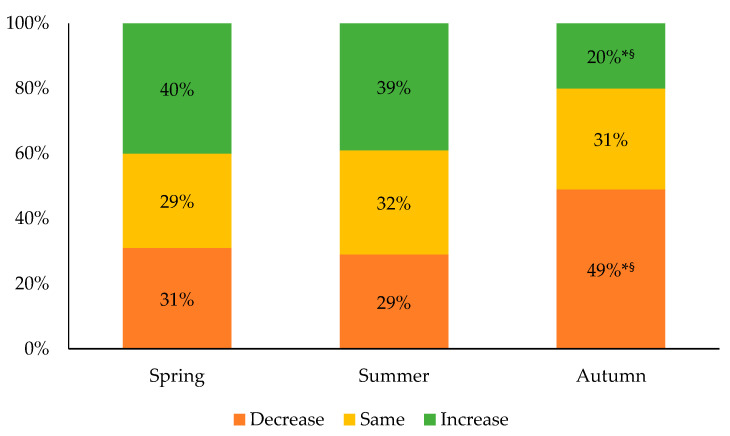
Evolution of sleep duration compared to the winter 2020. * Significantly different from the spring; ^§^ significantly different from the summer; *p* < 0.01.

The evolution of the sleep duration from the spring to the autumn in comparison to the winter is shown in Figure 4. During the spring, 40% of the participants reported an increase in their sleep duration of either a slight increase (17%) or a significant increase (23%). During that same period, 31% of the participants reported a decrease in their sleep duration, of either a slight decrease (13%) or a significant decrease (18%). In the summer, 39% of the participants had increased their sleep duration in comparison with the winter, reporting either a slight increase (20%) or a significant increase (19%). Many participants (29%) reported a decrease in their sleep duration during that same period, with 15% of them reporting a slight decrease and 14% a significant decrease. A significantly higher proportion of the participants (49%) reported a decrease in their sleep duration during the autumn compared to the winter of either a slight decrease (25%) or a significant decrease (24%). During that same period, 20% of the participants reported an increase in their sleep duration, with either a slight increase (13%) or a significant increase (7%). Again, a significant gender effect was observed for the three different periods, with a greater proportion of girls reported that they were experiencing changes in their sleep duration than boys or gender-diverse participants (all *p* < 0.001).

**Figure 5:**
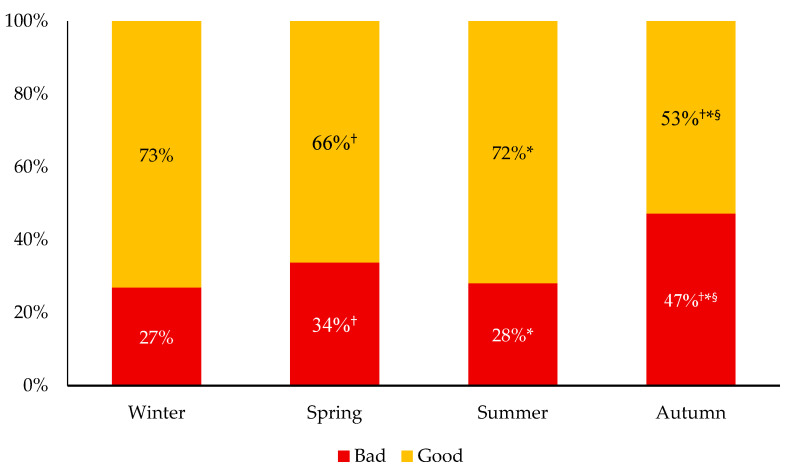
Evolution of sleep quality from the winter 2020 to the autumn 2020. ^†^ Significantly different from the winter; * significantly different from the spring; ^§^ significantly different from the summer; *p* < 0.001.

Moreover, the evolution of the sleep quality through the 2020 year is shown in Figure 5. That is, during the winter, 73% of the participants had experienced a rather good (55%) or a very good (18%) sleep quality, while 27% of them had experienced a rather bad (19%) or very bad sleep quality (8%). In the spring, 66% of the participants reported a rather good (38%) or a very good (28%) sleep quality, while 34% of them reported a rather bad (20%) or a very bad (14%) sleep quality. Most of the participants (72%) indicated that their sleep quality was rather good (40%) or very good (32%) during the summer. For the same period, a rather bad (17%) or a very bad (11%) sleep quality was reported by 28% of them. Finally, the sleep quality was at its lower level of the year during the autumn. Indeed, 47% of the participants had experienced bad sleep quality in the autumn, with 28% of them reporting a rather bad and 19% reporting a very bad sleep quality. During that same period, 39% of the participants reported a rather good and 14% reported a very good sleep quality, for a total of 53% of the participants that had experienced good sleep quality in the autumn. Compared to the winter, decreases in sleep quality were more commonly reported during the autumn than during the spring (*p* < 0.001). A greater proportion of gender-diverse participants had experienced bad quality sleep than girls or boys for these four different periods (*p* < 0.001).

### 3.2. Meeting the 24 h Movement Recommendations

The proportion of participants meeting each of the three 24 h movement behavior guidelines as well as the estimation of the proportion of adolescents following them as of December 2020 are presented in Table 1. On weekdays, 1.1% of the participants followed the recommendations for PA practice in their recreational screen time as well as for sleep duration. On weekends, this proportion was 1.4%. Participants meeting all of the three movement behavior recommendations on both weekdays and weekends represent 0.2% of the sample. Moreover, 50.0% of the participants reported meeting none of the three recommendations on weekdays and 41.7% on the weekend. That is, 26.3% of the participants reported meeting none of these recommendations, whether on weekdays or on weekends. It should be noted that 37.1% of the participants reported practicing less than 20 min of PA per weekday, compared to 41.2% per weekend day.

**Figure 6:**
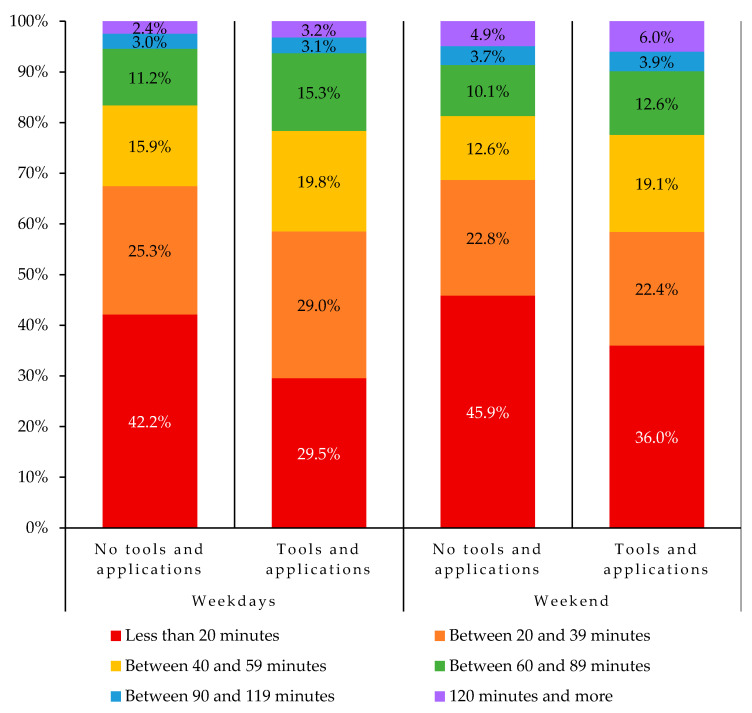
Association between the use of physical activity-related tools and applications and the daily physical activity in adolescents. Pearson chi square *p* value < 0.001 for this association on both weekdays and weekend.

**Figure 7:**
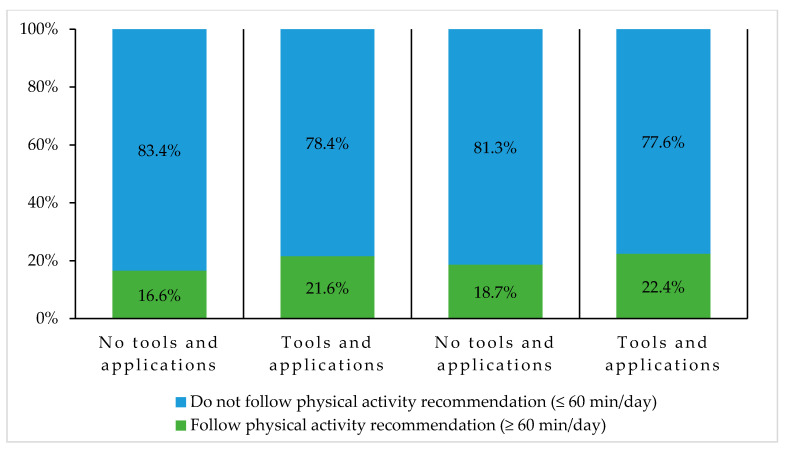
Association between the use of physical activity-related tools and applications, and the respect of the physical activity recommendation in adolescents. Pearson chi square *p* value < 0.05 for this association on both weekdays and weekend.

Regarding the use of technology while practicing PA, 40.8% of the participants reported using a tool or an application over the last month. An association between the use of a PA-related technology and the actual PA level of the participants was observed (*p* < 0.001), as shown in Figure 6. That is, lower proportions of participants using those types of tools or applications reported practicing less than 20 min of PA per day, for both during the week and the weekend, compared to other participants. Moreover, higher proportions of participants who used a PA-related tool or application reported meeting the PA recommendation, for both the week and weekend, compared to other participants (see Figure 7). The most popular categories of PA tools and applications were exercise inspiration (22.6%), tracking (18.9%), training exercises (14.1%), and watches (11.9%). Participants indicated using these technologies to track their PA (46.9%), to track their progress (32.1%), to find suggestions of new exercises (28.4%), to pass the time when they were bored (25.9%), and to count the calories burned while exercising (19.5%).

The proportions of participants reporting more than 4 h per day of recreational screen time on weekdays and weekends were 37.4% and 58.1%, respectively. Concerning sleep duration, 34.4% of the participants reported sleeping less than 7 h per night on weekdays, in comparison to 20.3% on the weekend. Furthermore, when compared to the results of a Canadian study performed before the COVID-19 pandemic [2], there were significantly less adolescents meeting the three 24 h movement behavior guidelines during the autumn 2020 than prior to the lockdown, whether taken two by two or all together (all *p* < 0.001). For example, it was observed in 2017 that 5.5% of a sample of 1126 12- to 17-year-old Canadians met all three recommendations, while 17.1% met none of them [2]. When taken individually, there were also less adolescents meeting the PA and the sleep duration recommendations in 2020 compared to 2017 (both *p* < 0.001), but no difference was observed for the screen time (*p* = 0.24). That is, there were respectively 24.4%, 68.1%, and 28.1% of adolescents meeting the PA, sleep duration, and screen time recommendations prior the COVID-19 pandemic. Finally, our results indicate that less than 0.5% (95% CI [0.000–0.004]) of adolescents met the 24 h movement behavior guidelines as of December 2020.

## 4. Discussion

### 4.1. Evolution of the 24 h Movement Behaviors

The present study aimed to firstly draw the evolution from February 2020 to December 2020 of the PA practice, recreational screen time, and sleep habits of adolescents. Our results indicate that the 24 h movement behaviors of the participants varied across the different seasons, and that these variations are consistent with the restrictive measures implemented by the Canadian governments for PA practice and recreational screen time. That is, compared to winter, a decrease in PA practice as well as an increase in recreational screen time were observed during the spring for more than half of the participants. These results are consistent with previous results [4,8,9] and seem to be directly associated with the imposed lockdown. During the summer, while restrictive measures were loosened which allowed organized sports to resume, an improvement in PA practice and recreational screen time compared to during the spring was observed. During the autumn, in comparison with the summer, the PA practice had decreased again, while the recreational screen time had remained similar. That is, in autumn, 40% of the participants were still experiencing a decrease in their PA practice compared to the winter, while 48% of the participants were still using screens for recreational use more often than during the winter. Severe restrictive measures were imposed in autumn, including hybrid learning at school for older students, organized sports interruption, and social distancing. Results of the present study indicate that a large proportion of adolescents were unable to adapt their PA practice and recreational screen time habits to get back to their usual behaviors in terms of time per day, while having to comply with the restrictive measures imposed due to the COVID-19 pandemic. However, it should be noted that the variations observed in adolescents’ behaviors may be partly due to the change across seasons itself, and, therefore, could have been partially observed independently of the COVID-19 pandemic. Strategies leading to movement behaviors changes during these exceptional times should be immediately implemented or enhanced, in order to favor the adoption of a structured day in line with the 24 h movement guidelines, such as street closures to allow PA practice while respecting social distancing, parental education about breaking up sedentary behavior, PA challenges with family and neighborhood, and so on [8].

Interestingly, the evolution of the sleep quantity and quality of the participants from the winter to the autumn 2020 differs from their PA practice and recreational screen time evolution. That is, it was observed that a larger proportion of adolescents had increased their sleep duration during the spring. This is in line with previous studies reporting more time sleeping in adolescents during the spring lockdown [4,10,11], and could be explained by the school closures, allowing adolescents to sleep longer in the morning. Moreover, no differences between the summer and the spring were observed in sleep duration. However, nearly half of the participants reported a decrease in their sleep duration during the autumn compared to the winter. A possible explanation could be a delayed sleep timing which may be caused by the unscheduled sleep (i.e., no specific bed or wake time) experienced during the spring and the summer 2020 [8]. The PA practice decrease and the recreational screen time increase may also be part of the explanation, as they are both associated with poorer sleep [12,13,14]. Thus, results of the present study support the ‘Structured Days Hypothesis’ [15], which claims that the structured environment usually offered by school helps students to adopt more healthy behaviors, including PA, recreational screen time, and sleep patterns. Furthermore, sleep quality was mostly altered during the autumn, with 47% of the participants reporting bad quality sleep compared to 27% in the winter, 34% in the spring, and 28% in the summer. That is, results of the present study suggest that the COVID-19 pandemic direct and indirect effects may have impacted sleep duration and quality in adolescents, and that these effects seemed to arise several months after the beginning of the pandemic, which might be underestimated to this day.

### 4.2. Meeting the 24 h Movement Recommendations

This study also aimed to estimate the PA levels, recreational screen time, and sleep duration of adolescents as of December 2020. That is, our results indicate that less than 1% of adolescents was able to follow the three recommendations of the 24 h movement guidelines on both weekdays and weekends. Moreover, 26.3% of the participants failed to meet a single recommendation on weekdays or on weekends, and this proportion rises to 50.0% when considering weekdays only. These results are in line with previous Canadian studies conducted during the spring lockdown [3,4], and suggest that no improvement was made regarding the 24 h movement behaviors in adolescents between April 2020 and December 2020. The sustained reduction observed in the healthy movement behaviors are certainly critical and might lead to important adverse impacts on adolescents’ development [13]. Therefore, substantial effort is required to promote healthy movement behaviors and to provide resources to support the adolescents, their parents, the secondary school stakeholders, as well as the community.

Furthermore, as adolescents already struggled to follow the 24 h movement guidelines prior to the COVID-19 pandemic [2,3], our results suggest that the practice of PA in adolescents depends to a large extent on organized PA, and that they have little autonomy. Based on these results, immediate solutions are needed to favor the adolescents’ PA practice despite the restrictions in place. That is, our results indicated that PA-related tools and applications could be an avenue to explore in this context, to enhance PA participation in adolescents. Previous studies reported similar findings among adolescents and adults [5,6]. Moreover, as adolescents’ trips outside the home are still almost exclusively to go to school, exceptional measures favoring the adoption of a healthy and physically active lifestyle should be implemented in secondary schools as soon as possible. For example, flexible school schedules allowing adolescents to practice PAs within both their in-class and at-home school days, as well as the prescription of home-based exercise programs could be implemented [16]. Furthermore, our results indicated that a substantial proportion of adolescents had perceived an increase in their PA practice despite the restrictive measures in place, such as the interruption of organized sports. This result raises questions on what happened with these adolescents, as well as on how actual organized sports, including at-school activities, ensure that they meet all adolescents’ needs in terms of PA. Further studies should consider examining these questions, to help find more solutions. Outdoor activities should also be encouraged in both in-school and at-home settings in order to favor PA practice [13,17]. Finally, measures aiming to increase the autonomy of all adolescents concerning the adoption of a healthy and physically active lifestyle, such as physical education and health classes, should be magnified, and new measures should be developed to counteract the actual situation [18,19].

### 4.3. Limitations

There were some limitations in the present study. First, our findings are limited to a population of students from public secondary schools in Montreal, Canada. Nonetheless, our results are strengthened by studying a homogenous population in a very large sample size. Second, as the participants completed the questionnaire in December 2020 and were asked to compare their behavior during specified periods of 2020 with their behavior prior to the onset of the COVID-19 pandemic, their answers may be subject to recall biases. Therefore, results of the present study should be interpreted with caution. Finally, another limitation was the use of self-reported measures of lifestyle habits. However, as the use of self-reported measures is usually associated with the social desirability bias [20], it is even more worrisome to observe these catastrophic results.

## 5. Conclusions

Results of the present study suggest that the restrictive measures due to the COVID-19 pandemic contributed to the worsening of the already worrying situation of the 24 h movement behaviors in adolescents, making it harmful for the adolescent’s health and development. Indeed, significant proportions of adolescents practice less PA, spend more recreational time on screens, and experience poorer sleep than prior to the spring lockdown. Moreover, as of December 2020, nearly none of the adolescents met the three recommendations of the 24 h movement guidelines, becoming a critical issue. To prevent the appearance of developmental problems in adolescents, policymakers, educators, families as well as the community should immediately take strong and direct actions to favor the adoption of a healthy and physically active lifestyle in adolescents during and after the COVID-19 pandemic.

## Figures and Tables

**Table 1:** Proportion of adolescents meeting the physical activity, recreational screen time, and sleep duration recommendations as of December 2020.

24 h Movement Behavior Recommendations	Weekdays				Weekend			
	All Students *n* = 2661	Girls *n* = 1417	Boys *n* = 1154	Gender-Diverse *n* = 33	All Students *n* = 2661	Girls *n* = 1417	Boys *n* = 1154	Gender-Diverse *n* = 33
**Specific recommendations**								
Physical activity (≥60 min/day)	18.3 [16.8–19.8]	15.8 [13.9–17.7]	21.2 [18.8–23.6]	28.1 ^§^ [12.5–43.7]	20.1 [18.6–21.6]	15.2 [13.3–17.1]	25.9 [23.3–28.5]	34.4 ^§^ [17.9–50.9]
Recreational screen time (≤2 h/day)	27.4 [25.6–29.2]	25.0 [22.7–27.3]	31.0 [28.2–33.8]	13.3 ^§^ [11.5–25.5]	14.0 [12.6–15.4]	13.2 [11.4–15.0]	15.0 [12.8–17.2]	6.7 [0.0–15.6]
Sleep duration (8 to 11 h/night *)	18.4 [16.9–19.9]	15.2 [13.3–17.1]	23.4 [20.8–26.0]	6.5 ^§^ [0.0–15.2]	39.4 [37.5–41.3]	38.1 [35.5–40.7]	42.2 [39.2–45.2]	29.0 ^§^ [13.0–45.0]
**Combination of recommendations**								
Physical activity and recreational screen time	5.0 [4.2–5.8]	3.8 [2.8–4.8]	6.5 [5.1–7.9]	6.1 ^§^ [0.0–14.5]	3.0 [2.3–3.7]	1.7 [1.0–2.4]	4.5 [3.3–5.7]	3.0 ^§^ [0.0–8.8]
Physical activity and sleep duration	3.4 [2.7–4.1]	1.8 [1.1–2.5]	5.3 [4.0–6.6]	3.0 ^§^ [0.0–8.8]	7.8 [6.8–8.8]	5.9 [4.7–7.1]	10.5 [8.7–12.3]	6.1 ^§^ [0.0–14.3]
Recreational screen time and sleep duration	6.1 [5.2–7.0]	4.8 [3.7–5.9]	8.2 [6.5–9.9]	0.0 ^§^ [0.0–2.9]	5.1 [4.2–6.0]	4.4 [3.3–5.5]	6.3 [4.8–7.8]	0.0 ^§^ [0.0–2.9]
All three recommendations	1.1 [0.7–1.5]	0.5 [0.1–0.9]	1.9 [1.1–2.7]	0.0 ^§^ [0.0–2.9]	1.4 [1.0–1.8]	0.6 [0.2–1.0]	2.5 ^§^ [1.6–3.4]	0.0 ^§^ [0.0–2.9]
None of the recommendations	50.0 [48.0–52.0]	54.0 [51.2–56.8]	44.0 [40.9–47.1]	57.1 ^§^ [38.8–75.4]	41.7 [39.7–43.7]	45.0 [42.2–47.8]	37.1 [34.1–40.1]	35.7 ^§^ [18.0–53.4]

Results are percentages [95% confidence interval]. ^§^ Denotes significant gender effect. * For 12–13 years old, recommendation is to sleep steadily between 9 to 11 h per night, while for 14–17 years old, it is to sleep steadily between 8 to 10 h per night. It should be noted that the different behavior recommendations’ categories are not mutually exclusive and that 57 participants did not indicate their gender.

## Data Availability

The data presented in this study are available on request from the corresponding author. The data are not publicly available due to privacy and ethical considerations.
